# Cost-effectiveness analysis of a chronic back pain multidisciplinary biopsychosocial rehabilitation (MBR) compared to standard care for privately insured in Germany

**DOI:** 10.1186/s12913-021-07337-9

**Published:** 2021-12-24

**Authors:** M. Hochheim, P. Ramm, M. Wunderlich, V. Amelung

**Affiliations:** 1 Institute of Epidemiology, Social Medicine and Health System Research, Medizinische Hochschule Hannover (MHH), Carl-Neuberg-Straße 1, 30625 Hannover, Germany; 2 Generali Health Solutions GmbH (GHS), Hansaring 40 – 50, 50670 Köln, Germany

**Keywords:** Humans, Low Back Pain, Cost-Benefit Analysis, Propensity Score, Sick Leave, Insurance, Health, Exercise, Mentoring, Germany

## Abstract

**Background:**

Multidisciplinary biopsychosocial rehabilitation (MBR) is highly recommended for chronic lower back pain (CLBP) treatment, but its economic benefit remains to be clearly demonstrated. The purpose of this study is to analyse the effect of a 12-month MBR programme of behavioural change coaching and device-supported exercise on direct medical costs, sick leave and health-related quality of life (HRQOL) at 24 months.

**Methods:**

An incremental cost-effectiveness analysis was conducted in Germany from a private health insurance perspective using data from a multi-centre, two-arm randomised controlled trial with parallel-group Zelen's randomisation and 24-month follow-up. After removing dissimilarities in characteristics between MBR and usual care (control) via propensity score matching, treatment effects were calculated using a difference-in-difference approach.

**Results:**

Base-case analysis of the MBR (*n*=112) and usual care group (*n*=111) showed an incremental cost-effectiveness ratio (ICER) of €8,296 per quality-adjusted life year (QALY) gained, indicating that the intervention was cost-effective. Compared to the controls, MBR reduced economically unaccounted sick leave due to back pain in the last six months by 17.5 days (*p* = 0.001) and had a positive effect on health-related quality of life (HRQOL) (0.046, *p*=0.026). Subgroup analysis of participants with major impairment demonstrated that a dominant intervention was possible, as reflected by an ICER of - €7,302 per QALY. Savings were driven by a - €1,824 reduction in back pain-specific costs. Moreover, sick leave was 27 days (*p* = 0.006) less in the MBR group.

**Conclusions:**

This first cost-effectiveness study with combined data from a private health insurer and a controlled trial in Germany demonstrated that long term MBR for the treatment of CLBP is cost-effective. Subgroups with major impairment from back pain benefitted more from the intervention than those with minor impairment. MBR significantly reduced sick leave in all participants. Hence, it is a profitable intervention from a societal point of view.

**Trial registration:**

The trial of the evaluation study was retrospectively registered in the German Clinical Trials Register under trial number DRKS00015463 retrospectively (dated 4 Sept 2018).

## Background

Lower back pain is the leading cause of years lived with disability (YLD) [1] as well as a frequent cause of absences from work and severe pain-related disability worldwide. Back pain places a high direct and indirect burden on health care systems and societies [2]. Estimates range from AU$9.17 billion per year in Australia to $91 billion per year in the USA [3, 4]. Germany lies in the middle, with up to €49 billion per year [5].

Chronic lower back pain (CLBP) is an urgent global public health concern [6]. It is often conceptualised as a biopsychosocial problem, i.e., as a complex and dynamic interaction between physical, psychological and social elements [7]. Multidisciplinary biopsychosocial rehabilitation (MBR) – a combination of physical, behavioural and/or social components, is recommended in clinical treatment guidelines for CLBP [8].

However, the actual care provided often does not correspond to clinical recommendations. Frequently, there is an oversupply of low-value care and an undersupply of high-value care. Passive therapies (e.g., massage or MRI) associated with high costs and little benefit are frequently provided [8]. Even if guideline-compliant treatment is carried out, it is unclear whether it is cost-effective [9]. Studies focusing on the cost-benefit of medium to long-term standardised treatment programmes for non-specific back pain are very diverse in terms of the types of treatments (*physical therapy, information & education, manual therapy, combined physical & psychological interventions*), comparators (*outpatient physiotherapy, usual care, usual care & component, self-care advice*), follow-up periods (*3 to 120 months*), stakeholder perspectives (*individual, society, health care provider*), cost information sources (*administrative vs. patient self-reported*) and target groups (*acute vs. chronic*) investigated [10, 11].

Herman et al. [12, 13] attempted to resolve this diversity by building a Markov model for the comparative analysis of usual care versus 27 alternative interventions in terms of their treatment costs, back pain-specific healthcare costs, productivity costs, total costs for society and providers, and quality-adjusted life years (QALY). The observed QALY gain compared to usual care across all programmes was 0.017. Active training interventions led to the highest QALY gains (0.033 for flexion-distraction [14] and active trunk exercise [14], respectively, and 0.048 for yoga [15]). Traditional Chinese medicine-based acupuncture [16] and spinal manipulation [17] yielded the lowest QALY gain (0.004 in each case). The mean incremental cost-effectiveness ratio (ICER) was $3,591 per QALY. Sixteen interventions were classified as “dominant”, i.e., associated with lower back pain-specific costs and higher benefits than standard care. Yoga also had the best cost-benefit ratio, characterised by a $1,136 reduction of back pain-specific costs with a simultaneous QALY gain of 0.048 [15]. Spinal manipulation [18] had the worst cost-benefit ratio; it achieved a slight QALY gain of 0.004 at an additional cost of $457 and an ICER of $114,250. The only MBR intervention included in their analysis achieved [19] a QALY gain of 0.015 in combination with a cost reduction of $172 and an ICER of - $11,466.

However, the results must be interpreted cautiously because these ICER estimates include only back pain-specific costs and not all direct costs. CLBP is a multidisciplinary biopsychosocial problem and, as such, its various causes are often associated with psychological disorders, multiple medical issues, such as obesity, smoking and lack of exercise [18, 20]. Therefore, the total direct costs of CLBP should be determined in cost-effectiveness analysis (CEA).

The few studies that used total direct health care costs found ICERs of £2,411 to AU$19,036 [21–23]. According to existing systematic reviews, more high-quality, site-specific studies are needed to reduce the uncertainty of cost effects for back pain treatments; moreover, these studies should have observation periods longer than 12 months and should use high reporting standards [9–11, 24]. Overall, evidence on the cost-effectiveness of structured long-term treatments is still so scarce, that it has been rated as a research priority [25].

Standardised, guideline-based disease management programmes (DMPs) have already been developed, implemented in the German health system, and evaluated for some chronic diseases (e.g. COPD), but not for CLBP [26]. Some insurance companies have developed and implemented different approaches, but they have rarely been evaluated scientifically [27, 28]. Currently, no published CEA data for any German long-term MBR programme for back pain exists.

This paper aims to close this gap. Based on the Consolidated Health Economic Evaluation Reporting Standards (CHEERS) criteria, it analyses whether a 12-month outpatient MBR intervention consisting of behavioural-change coaching and device-supported exercise with low entrance barriers is cost-effective compared to usual care in CLBP patients treated in a private health insurance setting in Germany [29].

Generali Deutschland Krankenversicherung AG, a German private health insurance company formerly known as "Central Krankenversicherung", launched a one-year MBR programme for chronic LBP in 2014. Hüppe et al. studied the feasibility and long-term efficacy of the intervention in a randomised controlled trial designed using Zelen's randomisation approach (German Clinical Trials Register registration number DRKS00015463). Their results showed the medical effectiveness of MBR over the 12-month follow-up [28, 30].

The rationale for the present study is to determine whether the MBR intervention is also cost-effective. In view of the lack of evidence-based data, our aim was to analyse and present data on the effect of a structured MBR programme for CLBP on direct total costs, back pain-specific costs and sick leave due to back pain. We thereby addressed the limitations of existing studies in numerous ways. First, we analysed cost data over a period of 48 months, which represents a long observation period. Second, we present data on the incremental costs and effects of back pain-specific MBR and total cost differences in such a way that the study can be compared with both arms of the existing data in the literature. Third, we measured the causal effect of MBR in a multi-site, real-world setting using a propensity score matching difference-in-difference (PSM-DiD) model [31]. Finally, this is the first cost-effectiveness study of a back pain-specific MBR programme that was conducted in Germany in accordance with the CHEERS statement.

The present CEA gives health care decision-makers an additional information tool for implementing and planning the content and duration of future DMPs for the statutory health insurance system in Germany.

## Methods

### Target population, subgroups and time horizon

The present economic evaluation is based on data from a two-arm controlled trial with a parallel-group Zelen’s randomisation design and supplemented with administrative direct health costs data. The original study had a 24-month observation period [28]. Study participants were recruited from April to October 2015. The cut-off for measurement of effectiveness ended with the two-year follow-visit, conducted between April and October 2017.

The trial was conducted in collaboration with Generali Deutschland Krankenversicherung AG, one of the largest private health insurance providers in Germany. In 2019, Generali had 308,088 fully insured members and 1,431,522 with supplementary partial insurance [32]. Only fully insured members were eligible to participate in the evaluated health intervention. Generali’s database was searched for administrative identification of adults (minimum age of 18) with CLBP based on their insurance billing data. Patients included in the study needed at least two cases of ICD-10 codes M40-M54 (dorsopathies) in combination with either a) temporary work disability due to back pain or b) opioid prescriptions or c) mental disorders within the last 12 months. Insurees who were unable to provide informed consent, unable to receive the intervention (e.g., due to physical or psychological impairment or to inability to understand basic spoken or written German), suffering from a terminal illness and/or currently participating in an alternative self-management health intervention, were excluded. The full list of inclusion criteria and details regarding the trial design can be found elsewhere [28].

In Germany, privately insured patients generally belong to higher socio-economic classes and are self-employed persons, civil servants or employees with a salary above the compulsory insurance threshold (currently €64,350).

One of the main results of the previously run effectiveness analysis was that the effect of the intervention was highly dependent on the level of impairment due to back pain [28]. Therefore, participants of the present study were divided into subgroups based on their overall result in the *Chronic Pain Grade Questionnaire* score [33, 34]. Two parameters were used to classify back pain severity levels: the characteristic pain intensity (score 0-100), calculated as an average of the current, average and maximum pain intensity, and pain-related impairment (0-6 points), calculated from the number of impairment days and the extent of the impairment experienced in everyday life, leisure and work. This is reflected by the four hierarchical Graded Chronic Pain Scale (GCPS) grades: Grade I, low disability-low intensity; Grade II, low disability-high intensity; Grade III, high disability-moderately limiting; and Grade IV, high disability-severely limiting [33]. Here, back pain patients with GCPS Grades I and II were classified as having *minor impairment* (functional chronic pain), and those with Grades III and IV as having *major impairment (*dysfunctional chronic pain) due to back pain.

### Intervention and comparators

The intervention was a combination of care approaches: Back pain was treated by a multidisciplinary network of general practitioners, orthopaedic surgeons and pain therapists working according to the Cologne Research and Prevention Centre (FPZ) concept, and treatment was provided as close as possible to the patient's home [35]. This concept is based on a spine-stabilising musculature training programme developed at the German Sport University Cologne, which has been further advanced by the FPZ for the rehabilitation of patients with sub-acute and chronic back pain (for details, see http://www.fpz.de). Based on the results of a functional biomechanical analysis, a treatment plan consisting of up to 24 one-hour equipment-supported training units to build up the spine-stabilising musculature was designed and carried out at FPZ back centres. After completing the training programme, MBR participants were eligible to receive €100 twice for the use of further freely selectable health sport offers as an exercise bonus.

Each participant received personal telephone support from an external health coach provided by Thieme TeleCare. The coaching initially accompanied the therapy and was then followed up for six months; the aim of coaching was to support behavioural changes, thus contributing to the continuation of physical activitiy.

The total cost of training and coaching was €1,500 per participant.

The control group did not receive the above training or coaching services, but rather “usual care” according to current practice, as described in the recommendations of the *National Clinical Practice Guideline for Non-Specific Low Back Pain* [36]*.* The guideline recommends exercise and behavioural therapy (i.e., multimodal therapy) as the primary form of treatment for non-specific CLBP after the exclusion of so-called ”red flags” indicative of a specific, pathological cause and a more serious aetiology [37, 38]. However, considering the current lack of a German DMP for CLBP, the prescribed treatments vary depending on the treatment preferences of the attending physician and/or patient and are thus highly heterogeneous [39, 40]. Twenty-three percent of newly diagnosed CLBP patients in Germany receive guideline-compliant multimodal therapy in the first year of occurrence [41]. Usual care for CLBP can therefore be defined based on the care reality, i.e., the full spectrum of patient care practices used in the treatment of CLBP, which includes physiotherapy, multimodal therapy, passive measures, injections, pain killers, imaging, surgical procedures and psychotherapy, among others. Due to the heterogeneity and individualised nature of treatment practice throughout Germany on the one hand and to the different risk factors for nonspecific CLBP on the other, it is not possible to establish a more precise definition of usual care.

### Health outcomes, study perspective and discount rate

The present cost comparison covered a period of four years. The patient’s individual start in the study was defined as baseline (t-0), and the costs incurred 24 months before and 24 months after t-0 were compared. A follow-up period of 24 months was used to compare health developments with economic effects, i.e., by calculating incremental cost-effectiveness ratios based on QALYs. No further tracking of cost developments into the future was carried out, as no longer-term data on the individual health status of the participants was collected. Two different datasets were merged:

The dataset provided by the University of Lübeck contained the collected primary and secondary outcome data at baseline and at 24 months for participants who successfully completed the study. The primary outcome measure in the original trial was CLBP severity (including the number of sick days due to back pain), which was assessed using the GCPS scale and the HRQOL scores ascertained using the German Short Form 12 (SF-12) instrument [42]. Secondary outcomes were the risk of back pain chronification (measured using the Keele STarT Back Screening Tool), psychological distress (assessed with the Patient Health Questionnaire-4, PHQ-4), and the self-reported level of physical activity [43–45]. Participants completed identical online questionnaires (self-assessment) at home at baseline and two years thereafter [28].

Information about participants’ use of health care services was extracted from the dataset provided by the insurance company. The dataset contained longitudinal patient-level information on medical diagnoses, direct medical costs and healthcare utilisation between 2010 and 2017. The costs were divided into outpatient and inpatient costs. In order to achieve better comparability with the statutory system, costs for elective private insurance benefits (e.g., one or two-bed hospital room) and dental treatments were excluded from the analysis. Costs for the following were included: general hospital services, GP and specialist care, medicines, remedies, alternative practitioners (e.g., chiropractor), aids and private medical treatment. Back pain-related costs were identified as ICD-10 M40 to M54.9 diagnoses.

Regarding the reimbursement procedure, privately insured persons generally pay their health care bills up front, submit the bills to the insurance company afterwards, and receive reimbursements according to the contract of their insurance benefits plan. The present study population consisted of fully insured persons as well as recipients of governmental benefits. Therefore, this cost analysis was based on the billed amount instead of the amount of the refund paid by the insurance company. Thus, the actual costs listed on the health care bill were compared with each other without taking into account which payer (health insurance company, government or individual) reimbursed the costs.

The analysis was conducted from the payer's perspective. The primary output of the present study was a CEA based on their cost and effectiveness data. For QALY assessment, the EQ-5D value was calculated from the SF-12 using Lawrence's algorithm [46]. Following the recommendations of the German *Institute for Quality and Efficiency in Health Care* (IQWIG), the discount rate was set at 3 % per year [47]. Sensitivity analyses were performed for a discount rate of 0 % and 5 %, respectiveley. All costs were converted to 2020 Euros (€) using consumer price indices.

The secondary outcome was the change in days of disability due to back pain based on the patient’s response to the question *("Approximately how many days in the last six months were you unable to carry out your normal activities (work, school/study, housework) due to your back pain?")*. Since not every participant was entitled to daily sickness benefits, no monetary value was assigned to the days of sick leave.

Data management and statistical analysis were carried out using the software R 3.6.0 software [48], including the packages listed in the bibliography [49–56].

## Analytic methods

### Economic analysis

A CEA was conducted using a propensity score matching difference-in-difference (PSM-DiD) model [31]. Cost-effectiveness was calculated using the discounted mean differences in outcome (average treatment effect in the treated, ATT) and effect (EQ-5D) on a two-year horizon as well as the intervention cost of €1,500, which was not included in the calculation of the ATT. The exercise bonus, an optional benefit that not every participant took advantage of, was not included in the ICER calculations. To avoid the Keeler and Kretin paradoxon [57]*,* costs and effects were equally discounted using the following equation:

$$ICER=\frac{\left( ATT\ Total\ Cost\ discounted\right)+ Interventioncost\ }{\ \left(\left( Difference\ in\ EQ-5D\right)\ast 2\ years\right)\ast Discount}$$

Non-parametric bootstrapping (5,000 replicates) was used to construct the 95% confidence interval (CI) of mean estimates [58]. The uncertainty around the cost-effectiveness ratio was evaluated with the bootstrapped distributions of incremental mean costs and effects and the results were plotted on a cost-effectiveness plane. Furthermore, the probability that a treatment was the optimal choice was calculated at the different thresholds of willing to pay (WTP) per QALY gain for each intervention. Cost-effectiveness acceptability curves were plotted with the probabilities for a range of possible values (λ). Sensitivity and subgroup analyses were additionally performed to further evaluate the uncertainty of the CEA [59]. We also analysed how the results changed in response to (1) the exclusion of participants whose back pain did not improve, (2) to intention-to-treat (ITT) analysis and (3) the unmatched group of the original study.

### Difference-in-difference regression

The aim of using the DiD method is to estimate the average effect of treatment on the treated patient (ATT). Differences in cost over time between the MBR group and the control group were compared using a regression model with an interaction term between period and treatment (Y= β0 + β1*[Period] + β2*[Treatment] + β3*[Period*Treatment]). The outcomes in the baseline period were measured two years before the respective index date. The time-series dimension of the two-year baseline period was removed by comparing the values over two years to avoid biased standard errors due to serial correlation [60]. Thus, we generated a single value per outcome measure for the baseline and follow-up period was generated.

**Fig. 1:**
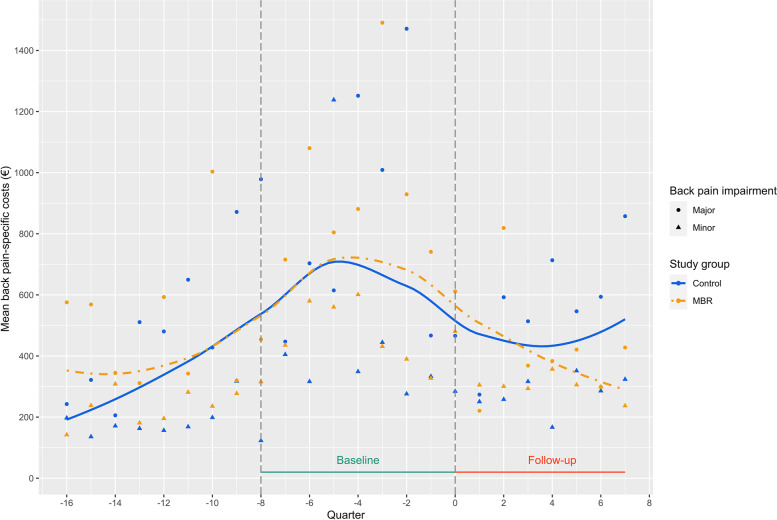
Parallel trend assumption showing the development of back pain-specific costs by back pain impairment and study group

To examine if the costs developed equally over time, parallel trend assumption [61] was used to plot back pain-specific costs over a four-year pre-treatment period. Outcomes were measured quarterly to test the parallel trend over 16 data points. The graph in Fig. 1 shows that the parallel trend assumption could be accepted. Thus, the back pain-specific costs in both groups developed similarly before the start of the intervention. The average pre-treatment costs could be divided by back pain impairment.

### Propensity score matching

A PSM-DiD model based on the one proposed by Heckman [31] was used to adjust for imbalances in the pre-treatment covariates (in the subgroups) imposed by Zelen’s randomisation approach. The DiD method provides unbiased effect estimates if the trend over time between the MBR group and the control group is identical in the absence of the intervention. However, due to the Zelen’s randomisation design and voluntary self-inclusion into the treatment arm, both groups may be incomparable, which could result in different trends over time. A nearest neighbour PSM with a caliper of 0.1 was performed to achieve adequate covariate balance at baseline [62]. Covariates used for the matching were sex, CCI, GCPS, STarT-Back, pre-treatment health costs, age, pre-treatment sick days and risk for CLBP, as identified in the participants’ selection [28].

As DiD regression is sensitive to high-leverage observations [63], extreme outliers (pre-treatment back pain-specific costs of at least €14,000) were excluded (*n*= 25). After excluding high-cost cases, the regression results became more robust.

### Analysis of covariance (ANCOVA)

A one-sided covariance analysis (ANCOVA) was carried out for the outcomes of secondary interest here, i.e. the reduction of sick days due to back pain and the general state of health (EQ-5D).

ANCOVA is a common, statistically robust method with model assumptions that should be respected (e.g. linearity between the covariate and the outcome, homogeneity of regression slopes, normally distributed outcome variable, homoscedasticity of residual variance) [64]. In the analysis of the EQ-5D, all model assumptions of the ANCOVA were met. For the range of sick days, however, the residuals were not normally distributed, but the effect of this was negligible as violations of these assumptions do not decisively influence either the probability of a first type error or the test strength [65, 66]. Covariance analyses are only contraindicated if the regression slopes are heterogeneous, the sample sizes are unequal, and the residuals are not normally distributed [67]. This was not the case here. Therefore, an ANCOVA can be applied.

## Results

### Characteristics of the study population

A total number of 189 participants in the MBR group and 254 participants in the control group took part in the reference study [28]. As the present study only included participants who completed the trial, there was no missing data on effectiveness. However, insurance data had to be processed in order to reach the final study size of the present work (Table 1). After data cleaning and matching, data sets for 112 participants in the MBR group and 111 in the control group were used for further analysis.

**Table 1:** Data preparation process for selection of the study population

Data processing steps and number of participants	Overall	MBR	Control	Used in
*Evaluation of the study group*	443	189	254	Published in [28]
*Exclusion of participants without any billing invoice available*	435	186	249	Sensitivity analysis III
*Exclusion due to unstable unit treatment*	431	185	246	
*Exclusion due to deductible/tariff*	404	172	232	
*Propensity score matching (ITT group) + truncation*	273	136	137	Sensitivity analysis II
*Exclusion of non-exercising participants*	223	112	111	Main analysis
*Analysis of participants who improved their back pain at the end of follow-up*	106	62	44	Sensitivity analysis I

Participants who did not participate in physical exercise training due to a large distance to the training centre were excluded from the baseline group (*n*=34) but included in the second sensitivity analysis^1^. Participants who dropped out during a later stage (during training or during coaching) were treated according to the intention to treat principle and kept in the study.

Four further participants had to be excluded based on the stable unit treatment value assumption for the estimation of treatment effects [68]: one participant from the MBR group was excluded as he enrolled himself in the programme a second time before the end of the follow-up period, and three control group participants had to be excluded as they enrolled themselves in the intervention before the end of the study period.

As the data was provided by a private health insurance provider, there was the additional obstacle of handling the insurance deductible, i.e., the yearly amount of health care costs a person must pay before their insurance starts to reimburse. To reduce the bias introduced by deductibles, the 27 participants who did not submit an invoice in one of the four examined years were excluded (yearly average amount of invoices = 42).

Table 2 shows the characteristics of the population before and after data processing and matching (see analytic methods). After processing, participants in the intervention group (MBR) and control group are almost equally distributed. Thus, diverging trends in the development of costs and effects were fixed and a more robust estimate of outcome differences between both groups could be achieved.

**Table 2:** Baseline characteristics of the intervention (MBR) and control groups before and after data processing

	Before			After		
	MBR	Control	***p***	MBR	Control	***P***
*n*	189	254		112	111	
*Age (mean (SD))*	53.86 (8.13)	54.14 (8.65)	0.725	55.68 (7.34)	54.69 (8.45)	0.354
*Gender = Female (%)*	70 (31.7)	103 (40.6)	0.072	39 (34.8)	40 (36.0)	0.960
*GCPS (%)*			0.062			0.948
*I*	64 (33.9)	115 (45.3)		41 (36.6)	42 (37.8)	
*II*	26 (13.8)	36 (14.2)		18 (16.1)	19 (17.1)	
*III*	52 (27.5)	49 (19.3)		28 (25.0)	24 (21.6)	
*IV*	47 (24.9)	54 (21.3)		25 (22.3)	26 (23.4)	
*STarT-Back (%)*			0.068			0.942
*1*	100 (52.9)	161 (63.4)		62 (55.4)	60 (54.1)	
*2*	64 (33.9)	71 (28.0)		36 (32.1)	28 (34.2)	
*3*	25 (13.2)	22 (8.7)		14 (12.5)	13 (11.7)	
*EQ-5D (mean (SD))*	0.61 (0.18)	0.63 (0.20)	0.253	0.61 (0.18)	0.60 (0.19)	0.78
*CCI*			0.661			0.6
*0*	76 (49.4)	127 (50)		56 (50)	47 (42.3)	
*1-2*	54 (35.1)	97 (38.2)		40 (35.7)	46 (41.4)	
*3-4*	20 (13.0)	23 (9.1)		14 (12.5)	14 (12.6)	
*>=5*	4 (2.6)	7 (2.8)		2 (1.8)	4 (3.6)	

Because the size and characteristics of the two groups were very similar at the beginning of the study period, the costs incurred could be properly compared. The population had a mean age of 55.19 years and a sex distribution of 35 % females and 65 % males. Moreover, 53 % of the study population had *minor* impairment and 47 % had *major* impairment due to back pain. Keele STarT Back Screening Tool assessment [43] showed that 55 % had a low, 33 % a medium and 12 % a high risk of persisting disabling symptoms.

Based on the weighted Charlson Comorbidity Index Score (CCI) method of classifying comorbidities that might alter the likelihood of mortality and/or high resource use [69], the groups were found to be comparable at baseline.

### Incremental costs and outcomes

The results varied slightly depending on which discount rate was applied. Since IQWIG recommends 3 % and the overall results did not change much, only the results of 3 % discounting are presented in the analysis. The changes in statistical significance due to discounting were marked. Table 3 presents the cost data for the 223 participants included in the analysis. Total medical costs were reduced in both the MBR and the control group, and the difference (-€780.61) was statistically non-significant (*p* = 0.78). Back pain-specific costs decreased in both groups, but were significantly higher in the intervention group than in the control group. The corresponding ATT was - €1156.83 (*p* = 0.039). The €754.98 decrease in inpatient costs was identified as the main driver of the reduction (*p* = 0.025). The number of sick days due to back pain in the last six months decreased with MBR, but increased in the control group. The estimated mean treatment difference was -17.5 days (*p* = 0.001) in the MBR group. EQ-5D development was more positive in the MBR group, compared to the control group (0.046, *p*= 0.026).

**Table 3:** Discounted outcomes for the intervention group (MBR) and control group in the baseline (2 years) and follow-up periods (2 years) with the respective difference-in-difference (DiD) estimator and its standard error (SE)

	MBR (n =111)		Control (n=112)		DiD Estimation	
Item	Baseline	Follow-up	Baseline	Follow-up	ATT^*a*^	SE
*Total cost (€)*	16630	14829	15837	14816	-781	2862
*Back pain-related cost (€)*	3454	2215	2748	2666	-1157*	570
*Inpatient cost – back pain (€)*	918	257	427	521	-755*	335
*Outpatient cost – back pain (€)*	2536	1958	2321	2145	-402	432
*Sick leave in last 6 months due to back pain*^*b*^	31.3	18.44	31.3	36	-17.5**	3.57
*Overall health status (EQ-5D)*^*b*^	0.604	0.678	0.604	0.632	0.046*	0.01

† < 0.1; * < 0.05; ** < 0.01; *** < 0.0001

^a^ Average treatment effect for the treated (ATT) represents the discounted mean differences in outcome

^b^ These results were not calculated with a DiD regression but with an ANCOVA

### Subgroup analysis

Back pain-specific costs generally vary depending on a patient’s GCPS grade: the higher the grade, the higher the costs [5]. To see if this applied to our dataset, the MBR and control groups were divided into two subgroups of participants with minor versus major impairment according to their back pain-specific impairment levels, as shown in Table 4.

**Table 4:** Difference-in-difference estimators (ATT) and their respective standard errors (SE) for analysis of two subgroups with (1) minor impairment due to back pain (GCPS Grades I and II) and (2) major impairment due to back pain (GCPS Grades III and IV), respectively, at study enrolment

**Minor impairment (1)**	**MBR (n =59)**		**Control (n=61)**		**DiD**	
**Item**	**Baseline**	**Follow-up**	**Baseline**	**Follow-up**	**ATT**^*a*^	**SE**
*Total cost (€)*	14636	15749	11242	11761	594	3564
*Back pain-specific cost (€)*	2935	2143	2177	1956	-571	701
*Inpatient cost – back pain (€)*	578	186	430	190	-152	348
*Outpatient cost – back pain (€)*	2356	1957	1747	1766	-419	566
*Sick leave in last 6 months due to back pain*^*b*^	3.3	5.8	3.3	15.3	-9.5*	2.91
*Overall health status (EQ-5D)*^*b*^	0.699	0.749	0.699	0.710	0.039	0.02
**Major impairment (2)**	**MBR (n=53)**		**Control (n=50)**		**DiD**	
**Item**	**Baseline**	**Follow-up**	**Baseline**	**Follow-up**	**ATT**^*a*^	**SE**
*Total Cost (€)*	18850	13805	21442	18543	-2146	4617
*Back pain-specific cost (€)*	4031	2296	3444	3533	-1824*	856
*Inpatient cost – back pain (€)*	1295	336	424	926	-1462*	600
*Outpatient cost – back pain (€)*	2736	1960	3021	2606	-362	633
*Sick leave in last 6 months due to back pain*^*b*^	64	33.2	64	60.4	-27.2**	7
*Overall health status (EQ-5D)*^*b*^	0.494	0.591	0.494	0.544	0.047	0.02

† < 0.1; * < 0.05; ** < 0.01; *** < 0.0001

^a^ Average treatment effect for the treated (ATT) represents the discounted mean differences in outcome

^b^ These results were not calculated with a DiD regression, but with an ANCOVA

Analysis of the *minor* impairment subgroup showed no significant difference between total medical costs (€593.61, *p* = 0.86) and back pain-specific costs (-€570.67, *p* = 0.42). The amount of sick leave was significantly lower in the MBR group than in the control group (-9.49, *p* = 0.024). The overall health status improved in both groups but did not reach the level of statistical significance (0.039, *p*= 0.119).

Analysis of the *major* impairment group revealed no significant difference in the overall direct cost (- €2,146.97, *p* = 0.64). However, the ATT for back pain-specific cost was significant, as reflected by a reduction of - €1,824.20 (*p*= 0.036) as a favourable effect of MBR, which was mainly driven by a reduction in hospital costs (- €1,462,34, *p* = 0.016). The difference in sick leave due to back pain in the last six months was 27 days (*p* = 0.006). The development of the overall health status improved by 0.047 points, but the difference was not significant (*p*= 0.158).

### Incremental cost-effectiveness ratio

The incremental cost-effectiveness ratio (ICER) was calculated using the discounted ATT and EQ-5D difference for the two years specified above as well as the intervention cost of €1,500. Based on the threshold of £20,000 to £30,000 per QALY set by the National Institute for Health and Care Excellence (NICE) [70], the intervention can be defined as cost-effective in view of its ICER of €8,296 per QALY gained. If one would be able to steer the participants better before enrolment and assign them to the appropriate subgroup, one could achieve an ICER of - €7,302 per QALY gained. In doing so, one would create a dominant intervention that saves costs and improves outcomes.

Calculation using only cost differences in back pain-specific costs reduced the ICER to €3,957 for the main group and to - €3,659 for the subgroup with major impaired subgroup.

**Fig. 2:**
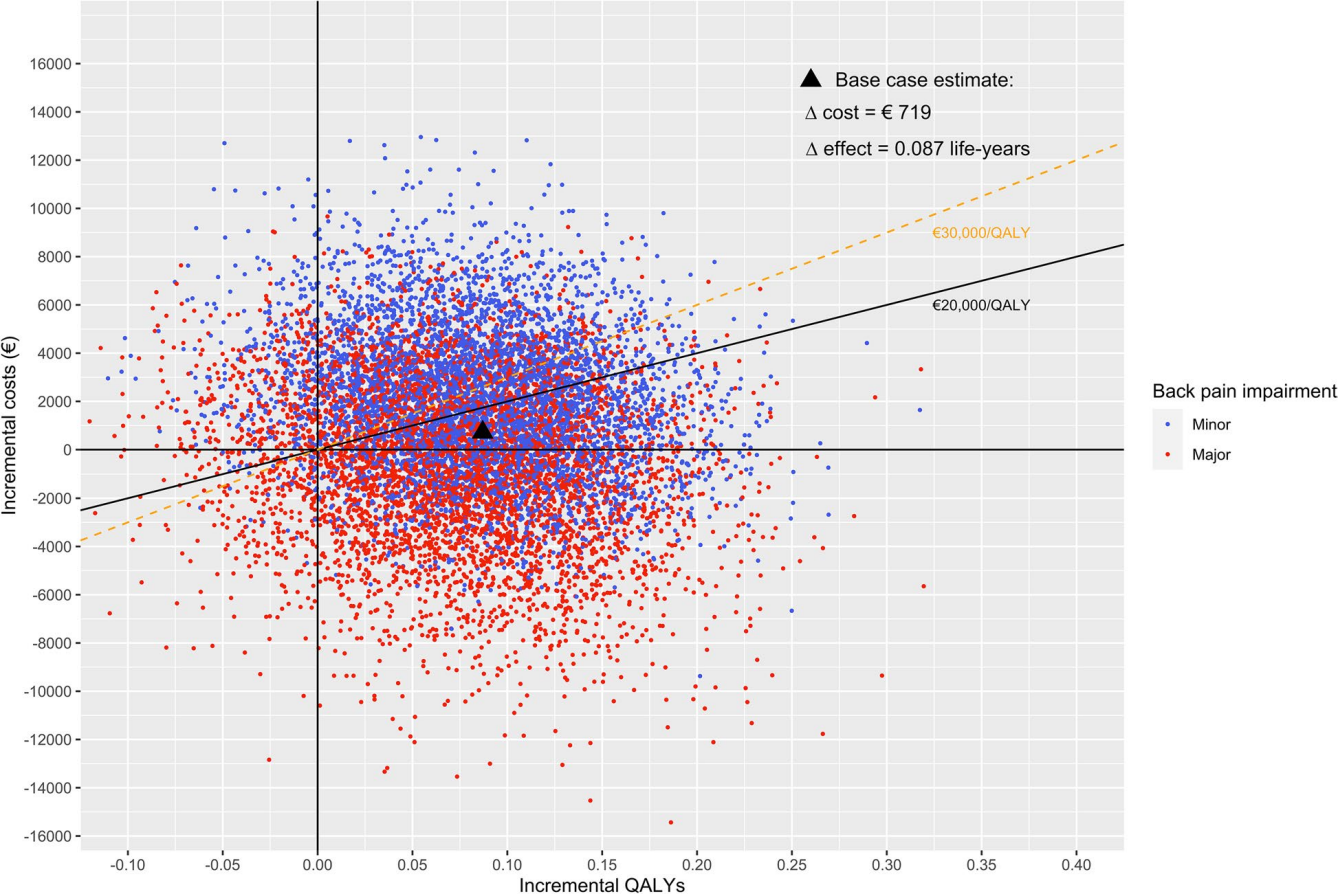
Scatter plot of incremental costs (in Euros) and incremental effects (in quality-adjusted life years, QALY) of MBR versus usual care, as determined by bootstrap resampling (base case), by level of back pain impairment (minor or major)

**Fig. 3:**
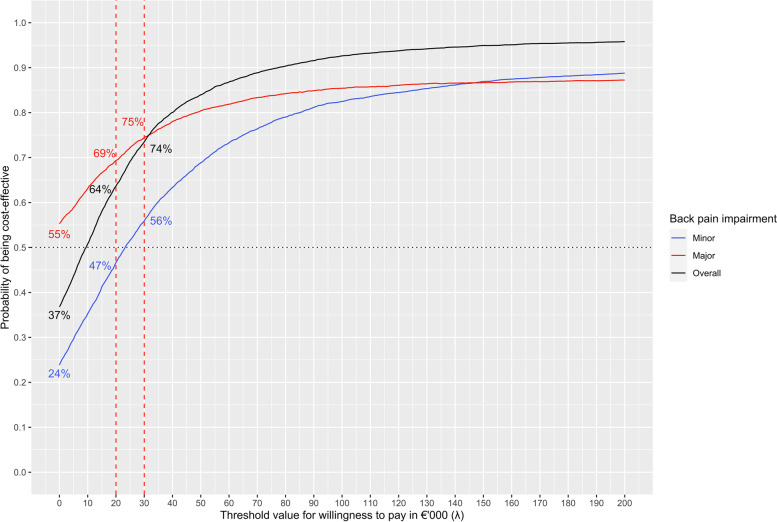
Cost-effectiveness acceptability curves for the probability of MBR versus usual care being cost-effective over a range of values for the maximum acceptable ceiling ratio (λ) by level of back pain-related impairment (minor, major, overall)

The bootstrapped results for MBR and usual care (control) cases were plotted on a cost-effectiveness plane and presented Figure 2. The majority of the bootstrapped replicates for the minor impairment cases (70 %) fell in the northeast quadrant of the cost-effectiveness plane, indicating a costlier and more effective intervention. The majority of bootstrapped replicates for the major impairment cases (50 %) fell in the southeast quadrant, indicating a less costly and more effective intervention. Nine percent of all replicates ended up on the left side of the Y axis, signalling a less effective intervention. Overall, the majority of bootstrapped replicates were below the willingness to pay (WTP) threshold. The cost-effectiveness acceptability curves (Fig. 3) showed that the estimate was robust. Overall, the probability that the intervention was cost-effective was 64% and 74% at a WTP threshold of €20,000 and €30,000 per QALY, respectively.

### Characterising uncertainty

One source of uncertainty was the back pain-specific cost, which could be accounted in case of specification of an ICD group M40 to M54 diagnosis on a submitted invoice. However, other diagnoses were often co-included on those invoices, resulting in ambivalence in cost allocation. In cases of doubt, it was not possible to distinguish precisely which costs were to be allocated to which diagnosis on the bill. This problem concerned MBR and control group participants equally and arose mainly in the outpatient sector. In order to compare whether cost-intensive co-diagnoses were more frequently coded in one group, the back pain-specific invoices were analysed additionally.

Table 5 shows how many invoices with ICD-10 M40-54 diagnoses were taken into account on average, how many invoices only contain an M40-54 diagnosis, and which comorbidity groups were most frequently listed. As can be seen in Table 5, the included bills were highly comparable to each other. The MBR group had, in median, two more bills containing an M diagnosis before the start of the intervention, but this difference diminished in follow-up.

**Table 5:** Characterising uncertainty of back pain-specific invoices by comparing median of back pain invoices and distribution and frequency of listed co-diagnoses at baseline and follow-up period

	MBR (*n* = 111)		Control (*n* = 112)	
Time	Baseline	Follow-up	Baseline	Follow-up
*Median*	10	9	8	8
*M40-54 Diagnosis alone*	33.2 %	27.4 %	31.1 %	27.6 %
*with other Diagnosis*	66.8 %	72.6 %	68.9 %	72.4 %
*Rank 1 ICD Group (%)*	M (25 %)	M (25 %)	M (21 %)	M (23 %)
*Rank 2 ICD Group (%)*	E (14%)	E (15 %)	E (11 %)	E (12 %)
*Rank 3 ICD Group (%)*	I (8%)	R (9 %)	R (10 %)	R (11%)
*Rank 4 ICD Group (%)*	G (8 %)	I (8%)	K (10 %)	K (10 %)
*Rank 5 ICD Group (%)*	R (8 %)	F (8 %)	I (9 %)	I (10 %)

ICD-10 codes:

E = Endocrine, nutritional and metabolic diseases

F = Mental and behavioural disorders

G = Diseases of the nervous system

I = Diseases of the circulatory system

K = Diseases of the digestive system

M = Diseases of the musculoskeletal system and connective tissue

R = Symptoms, signs and abnormal clinical and laboratory findings, not elsewhere classified

The main comorbidities in follow-up only differed from rank four onwards. The most commonly mentioned comorbidities were gonarthrosis in the group of other M diagnoses, diabetes mellitus in the E group, essential hypertension in the I group, other chronic pain in the R group, fatty liver in the K group, polyneuropathy in the G group, and unspecified depressive episodes in the F group.

Due to minor differences in the distribution of the M diagnoses and the co-mentioned groups, it could be assumed that the back pain-specific costs in both groups were not influenced by incorrect allocation.

### Characterising heterogeneity

The results of the main analysis indicated that participation in MBR could save costs in the long run. The reduction in back pain-specific costs was significant both in the overall population and in the subgroup with *major* impairment. Due to the large amount of standard deviation, the effect on total costs could not be clearly interpreted. In order to understand the development of the total costs better, we performed a first sensitivity analysis which included only those participants whose back pain improved, as reflected by a lower STarT-Back raw value accompanied by no regression on the GCPS ("profiteer" subgroup).

A second sensitivity analysis was conducted with all participants who enrolled in the programme, regardless of whether they finished all components or not (intention-to-treat subgroup, ITT). Explicitly, individuals who participated in the coaching offerings but not the physical training were also included in this analysis. As matching in itself was a possible source of bias due to the small sample size, a third sensitivity analysis was performed to compare the results obtained without PSM.

### Sensitivity analysis I: results of the outcome-based analysis

Table 6 presents the results of the first sensitivity analysis of “profiteers” whose back pain improved. Two years after the baseline measurement, back pain improved in 62 out of 111 (56 %) participants in the MBR group compared to only 44 out of 112 (39 %) in the usual care group (controls). The total medical costs of profiteers in the baseline period showed a range of €118,000. To exclude outliers, the groups were subdivided by low and high cost (0.1 – 0.5, 0.51 – 0.9 of the quantiles) members yielding 49 MBR and 34 control group profiteers with back pain improvement.

**Table 6:** Sensitivity analysis of participants whose back pain decreased, with the respective difference-in-difference (DiD) estimator (ATT) and its standard error (SE) in the baseline and follow-up period

**Low cost (0.1-0.5 quantile)**	**MBR (n =24)**		**Control (n=10)**		**DiD**	
**Item**	**Baseline**	**Follow-up**	**Baseline**	**Follow-up**	**ATT**^*a*^	**SE**
*Total cost (€)*	9699	10239	8821	6904	2457	1926
*Back pain-related cost (€)*	2054	1348	1369	1426	-763	790
*Inpatient cost – back pain (€)*	377	72	0	207	-512	470
*Outpatient cost – back pain (€)*	1676	1276	1369	1220	-250	677
*Sick leave in last 6 months due to back pain*^*b*^	32.8	8.8	33.8	14.5	**-**5.7	6.04
*Overall health status (EQ-5D)*^*b*^	0.607	0.745	0.607	0.634	0.111*	0.04
**High cost (0.5-0.9 quantile)**	**MBR (n=25)**		**Control (n=24)**		**DiD**	
**Item**	**Baseline**	**Follow-up**	**Baseline**	**Follow-up**	**ATT**^*a*^	**SE**
*Total cost (€)*	23749	13555	23420	19283	**-5787†**	3266
*Back pain-related cost (€)*	7588	3081	6292	4500	-2676	2130
*Inpatient cost – back pain (€)*	2700	0	2840	196	-56	1496
*Outpatient cost – back pain (€)*	4888	3081	3453	4264	**-2620†**	1519
*Sick leave in last 6 months due to back pain*^*b*^	44.45	9.42	44.35	27.73	-**18†**	6.7
*Overall health status (EQ-5D)*^*b*^	0.542	0.717	0.542	0.678	0.039	0.03

† < 0.1; * < 0.05; ** < 0.01; *** < 0.0001

^a^ Average treatment effect for the treated (ATT) represents the discounted mean differences in outcome

^b^ These results were not calculated with a DiD regression but with an ANCOVA

Analysis of the low-cost group revealed no significant difference in the costs between MBR and control profiteers. The only finding was a difference in improvement of the overall health status in favour of MBR (0.11, p = 0.034). The resulting ICER was €18,909 for the low-cost group.

In the high-cost group, MBR reduced the total costs by €5,787 (p = 0.08) compared to the control group (undiscounted: - €7,220, p = 0.04), corresponding to a dominant ICER (- €58,302).

The number of sick days decreased significantly in the low and high-cost groups; the decrease was greater with MBR than in the control group (low: -5.7, p = 0.44, high: - 18.3, p = 0.06). Statistical significance prevailed below a level of 0.1 in the high-cost group.

### Sensitivity analysis II: results Intention-to-treat

Table 7 shows the results of the second sensitivity analysis, which included all participants who enrolled in the programme, regardless of whether they finished all components according to protocol or not (ITT). The second sensitivity analysis revealed no significant difference in average treatment effect on costs or health. A significant reduction of sick leave due to back pain was observed. In the complete group, the MBR had an ATT of 13.6 days (p= 0.002). The decrease in sick leave due to back pain was 22 days more (p= 0.012) in the *major* impairment group (n=62) than in the control group (n=57) compared to only 7.2 days more (p=0.085) in the *minor* impairment (n=74) compared to the control group (n=80). The corresponding ICERs were €44,302 for the overall population, €99,039 for the minor impairment group, and - €5,559 for the major impairment group.

**Table 7:** Sensitivity analysis of two scenarios (intention-to-treat and original evaluation study), with the difference-in-difference (DiD) estimator (ATT) and its standard error (SE)

**Intention-to-treat (1)**	**Overall** ***n*= 136 (MBR) and 137 (controls)**		**Minor impairment** ***n*= 74 (MBR) and 80 (controls)**		**Major impairment** ***n*** **= 62 (MBR) and 57 (controls)**	
**Item**	**ATT**^*a*^	**SE**	**ATT**^*a*^	**SE**	**ATT**^*a*^	**SE**
*Total cost (€)*	755	2447	2981	2864	-1762	4249
*Back pain-related cost (€)*	-128	677	569	882	-959	1019
*Inpatient cost – back pain (€)*	231	535	971	697	-704	824
*Outpatient cost – back pain (€)*	-359	381	-402	498	-256	562
*Sick leave in last 6 months due to back pain*^*b*^	-13.58*	3.13	**-7.24†**	2.9	-22.04*	6.24
*Overall health status (EQ-5D)*^*b*^	0.027	0.01	0.024	0.02	0.025	0.02
**Original evaluation study (2)**	**Overall** ***n*= 186 (MBR) and 249 (controls)**		**Minor impairment** ***n*= 88 (MBR) and 147 (controls)**		**Major impairment** ***n*= 98 (MBR) and 102 (controls)**	
**Item**	**ATT**^*a*^	**SE**	**ATT**^*a*^	**SE**	**ATT**^*a*^	**SE**
*Total cost (€)*	2845	2365	**5330†**	2990	1217	3811
*Back pain-related cost (€)*	537	869	1022	1046	488	1451
*Inpatient cost – back pain (€)*	650	690	1078	825	540	1185
*Outpatient cost – back pain (€)*	-113	380	-56	517	-53	566
*Sick leave in last 6 months due to back pain*^*b*^	-12.7**	2.53	-4.68	9.42	-22.84**	4.95
*Overall health status (EQ-5D)*^*b*^	0.030*	0.01	0.011	0.01	0.048*	0.02

† < 0.1; * < 0.05; ** < 0.01; *** < 0.0001

^a^Average treatment effect for the treated (ATT) represents the discounted mean differences in outcome

^b^These results were not calculated with a DiD regression but with an ANCOVA

### Sensitivity analysis III: results without matching

Since the results and their significance level seemed to be dependent on the matching, we ran a third sensitivity analysis (second part of Table 7) with the original evaluation group [28]. The data preparation was omitted (no consideration of dropouts, deductibles, or high-cost cases). Only those participants with no billing information over the course of four years (n=8) were excluded. In the overall population and the *major* impairment group, there was no significant cost difference between programme participants and non-participants. In the control group, individuals with *minor* impairment had a favourable ATT of €5,329.67 (p = 0.064).

The health status and the number of sick showed a favourable effect of MBR. In the whole group, the ATT for sick days was nearly 13 days (p = 0.001), and the health gain was 0.03 (p = 0.039). In the *major* impairment group, participation in the health programme reduced sick days by almost 23 days (p = 0.001) and increased the health status score by 0.05 points (p = 0.030). The derived ICERs were €76,827 for the overall population, €329,361 for the minor impairment group and €30,026 for the major impairment group of the original evaluation study.

## Discussion

### Summary and comparison with prior work

This evaluation included real-world evidence gathered in the scope of a long-term outpatient chronic back pain-specific MBR programme providing behavioural change coaching and device-supported exercise to eligible members of a private health insurance company in Germany from 2013 to 2017. The findings demonstrated that the classification of participants according to their individual degree of impairment at the beginning of treatment based on their GCPS grade is a very good separator for effectiveness.

MBR achieved significant cost savings, especially in patients with *major* impairments: the isolated back pain-specific costs decreased by - €1,824. It should be noted that participation in the MBR programme requires at least one doctor visit before the start of the training because a medical prescription is required for FPZ training, and an MRI study is often ordered to rule out medical contraindications. Despite these extra charges, the MBR still resulted in a significant reduction of back pain-related costs. These findings indicate that the intervention has a long-term cost-saving effect. This is in line with the results of Müller et al. [71], who compared the cost-effectiveness of a multimodal back exercise programme for patients enrolled in Germany's statutory health insurance system with that of usual care. They found that the cost savings depended on the GCPS back pain grade at baseline, whereby the intervention was particularly cost-saving for participants with GCPS Grade IV. In contrast, the intervention examined here can be classified as an MBR with fewer training units but an additional coaching component that also focuses on behavioural change. We conclude that MBR can achieve back pain-specific cost savings in back pain patients with GCPS Grade III and higher.

With a QALY gain of 0.046, the intervention achieved the second-best benefit compared of all forms of therapy evaluated by Herman et al. [12]. One year of MBR resulted in high programme costs of €1,500, but achieved savings of €781 for total costs, and €1,157 for back pain-specific costs and an ICER of €8,296 per QALY gained (or €3,957 per QALY gained in case of back pain-specific costs only). As 91 % of the bootstrapped results fell on the right side of the Y axis, the intervention evaluated in this study can be classified as effective. In line with this, the cost-effectiveness acceptability curve shows that the probability that MBR is cost-effective is 64 % at a WTP threshold of €20,000, and 74 % at a threshold of €30,000 per QALY.

If health insurance companies were able to steer the participants better before enrolment and properly classify them as having minor or major impairment, a dominant intervention with an ICER of -€7,302 could be achieved. The majority of the bootstrapped replicates of patients with major impairment fell in the southeast quadrant, supporting the certainty of a dominant intervention. Herman et al. have shown that most interventions for CLBP are cost-effective from the perspective of the payer and that they are dominant for society [12]. Focus on the direct medical costs results in cost-effectiveness, but the additional consideration of the days of sick leave saved (-17.5 in the last six month) turns the intervention into a cost-saving instrument from the societal perspective. Furthermore, we showed that by consistently using the GCPS Grade to select patients for participation in the programme, it is possible to create a dominant intervention from the payer's point of view.

The results of this study revealed that back pain prevailed after two years in 61 % of participants in the control group, who received the usual care. This is consistent with the notion that back pain is a long-term condition with a variable course [72]. The exact timing of recurrence is unclear, but 33 % to 67 % of people with back pain can be expected to have permanent recurrent episodes [6]. The present study adds to the existing knowledge that if back pain is improved, the outcome differs strongly in terms of cost. The total costs were reduced in both of our "profiteer" groups. However, in the high-cost subgroup, MBR resulted in more savings in total costs than the usual care control group (- €5,787). The focus on exercise and self-efficacy reduced the high total cost significantly. From a purely cost-specific perspective, it can be stated that health insurance companies should increase their efforts to persuade severely impaired and cost-intensive back pain patients to participate in an MBR programme, and find alternative offerings for *minor* impairment and low-cost groups.

One possibility would be to offer a combination of yoga and behavioural change coaching for patients with minor impairment. In the Markov model by Herman et al., yoga had the best cost-benefit ratio; it was associated with a $1,136 reduction in back pain-specific costs and a simultaneous QALY gain of 0.048. Groessl et al. [21] also depict yoga as a promising solution. They investigated a 12-week-long progressive yoga intervention in a total of 150 participants and reported a QALY gain of 0.043 and an additional healthcare cost of $193 with a resulting ICER of $4,488. As yoga is a form of group exercise, it could be delivered for a fraction of cost of the investigated intervention. However, a huge barrier to yoga becoming a component of insurance company-based MBR programmes is that quality standards and forms of yoga are very diverse, which results in differential outcomes [73]. Unless teachers of effective yoga programmes are bundled in a nationwide network organisation (like FPZ), it will be difficult for insurance providers to guarantee certain exercise components. Another option would be to offer e-health or m-health programmes for the self-management of CLBP. Despite the current lack of data on long-term effectiveness and cost-effectiveness of such digital interventions, the published short- and mid-term data are promising [74, 75]. The Rise-uP trial compared a completely digital treatment consisting of electronic case reports, tele-consultations and a multidisciplinary mobile back pain app for all patients with usual care in Germany. At three-month follow-up, the intervention group had significantly greater pain reduction [76]. The cost-effectiveness and long-term effectiveness of this completely digital programme have yet to be proven, but should be monitored closely to find alternatives for the treatment of patients with minor impairment due to back pain.

However, if the perspective of society is also taken into account, participation in a back pain programme is worthwhile for all patients affected. Across all groups and sensitivity analyses, the reduction in sick days due to back pain was significantly higher in the MBR group than in the control group (ranging from 9.5 to 27 days). Wagner et al. reported a reduction of 44.3 days in the duration of incapacity to work and back pain-specific cost savings of €1,284 (daily sickness allowance excluded) after the completion of a 20-day course of short-term interdisciplinary multimodal pain therapy [77]. The effects were observed after one year; however, their programme is more time and cost-intensive (more than 100 hours of treatment and twice the expense). The target group is comparable to the major group, and the results are similar, even if the period under consideration is different. If one takes into account that there was a reduction of 27 sick days in the last six months, it can be assumed that a long-term MBR programme is not inferior to a focused, more expensive short-term interdisciplinary multimodal pain therapy programme in terms of sick days and cost reduction. The results observed in the *major* impairment group suggest that MBR can become superior in terms of access, cost-savings and reduction in sick leave if the provider finds a way to allocate patients to the intervention in a more targeted manner.

Sensitivity analyses II and III indicated that the payer should also aim to ensure that MBR programmes are carried out fully and analysed thoroughly with regard to cost-effectiveness. Privately insured patients are subject to special cost issues. First, they must not necessarily submit invoices for incurred costs and, second, the heterogeneity of the population with regard to the overall health burden on the other need to be taken into account. This underlies the need to conduct such a cost analysis in a large population that can be better and more clearly separated. Nevertheless, the sensitivity analyses performed in the present study confirmed the positive effect of the intervention on sick days and general health, which suggests that these effects are robust.

### Strengths, limitations and implications for further research

The strength of the present study is the combination of clinical trial data and real-world evidence from a health insurance database of settled claims. Thus, we were able to merge unbiased and clear cost data with medical outcome measures. Costs could be collected directly from the health insurance database and did not need to be estimated based on participant self-reports. This led to a very high internal validity, on the one hand, and to less time required for participants to fill out the study questionnaire on the other. However, study size had to be reduced for various reasons, and the data analytical options were limited. As described in Table 1, a total of 39 participants had to be excluded due to missing or unclear cost data. Due to the structure of the data set, the frequency of resource use also could not be clearly determined in some cases. The primary purpose of the private health insurance company’s database was to settle claims, which does not require the storage of information about the frequency of resource use. For example, a treatment such as physiotherapy may include ten therapy units, but only one invoice is submitted, billed and stored. The fact that information about the number of visits is not stored limits the number of analytical options. Even though the combination of real-world evidence and clinical trial data has been called the most powerful evidence-based research method in medicine [78], the aforementioned limitations should be considered in further research. For example, study participants could be additionally surveyed about their health service use or excluded from study participation in advance if they have a deductible.

Uncertainty also existed in the back pain-specific cost analysis due to the inclusion of co-diagnoses on the bill (see section on characterising uncertainty). By analysing the comorbidities in detail, we attempted to keep the influence of other diseases as low as possible. If residual doubt remains, this problem can only be remedied by focusing solely on inpatient visits due to back pain. Since a large proportion of the costs incurred in the outpatient setting and only a small number of the study participants were treated as inpatients, this was not done here in the interest of not reducing the sample size further. In case of a larger population, inclusion of inpatient costs only could be a feasible approach to the clarification of back pain-specific costs. It should also be noted that higher total costs are to be expected in the statutory health insurance (SHI) system. In contrast to private health insurance providers, SHI providers must pay sickness benefits after a certain period of absence from work. Therefore, the cost-effectiveness of long-term MBR treatment should also be researched in the statutory health insurance setting. This would prevent the situation of needing sample size reduction, as was the case here. Even though our sample size (443 participants) was larger than that of most other published studies [9], it was still challenging to present clear results. The sample size was reduced by excluding dropouts, treatment switchers, insurees with deductibles, truncation and PSM. In the course of the analysis, groups that were still cost heterogeneous had to be separated according to back pain status. The results of our analysis suggest that participation in MBR results in savings in many cases, but occasionally the results did not reach significance levels. Those findings should be verified in a larger study and, ideally, in a statutory health insurance setting where sick leave can be given a monetary value and more comprehensive cost and usage information should be available.

A PSM-DiD model was used to adjust for the imbalances in the pre-treatment covariates imposed by Zelen’s design randomisation. It successfully removed differences in distribution of back pain severity at baseline between MBR and the control group. However, in doing so, the group size was reduced and subgroup allocation changed substantially. The percentage of participants in the intervention group after data processing increased for GCPS Grades I and II and decreased for Grades III and IV. In the control group, it decreased for Grade I and increased for all other grades. The biggest movement occurred in the Grade I group. The MBR and control group contained 64 and 115 participants, respectively, before matching, and 41 and 42 after matching. Although intended to yield a similar distribution within all groups, matching resulted in the removal of a number of participants with low back pain severity and, therefore, low health care expenses. Sensitivity analysis III showed the effect of having a large proportion of Grade I back pain on cost development. If this imbalance had been kept in the dataset, the ICER for the minor impairment group would have increased to €300,026. The main CEA analysis was run with a smaller but more balanced number of cases. Although necessary here due to the parallel-trend assumption of the DiD and the goal of finding similar treatment groups that only differ in the level of exposure to the intervention, this should be avoided in future research. This situation could be contained by using an appropriate study design. As true randomisation should increase the chances of similar groups at baseline, we recommend this in further studies [79].

In the present study, the ICER was calculated without the exercise bonus for two main reasons: first, the exercise bonus was not a fixed cost of the intervention but rather a variable, optional “reward” that participants could receive on request. However, actual utilisation of the exercise bonus was limited, potentially due to the participants’ economic background. Second, the bonus was abolished by the insurance after the end of the trial due to high operational costs and low usage. Furthermore, the provider assumed that a take-away effect was taking place and therefore discontinued offering it. Even though the provider did not report any changes in participant behaviour without the exercise bonus, it must be noted that the evaluated intervention included it. The role that the bonus played in motivating participants to stay active is therefore unclear. However, if the intervention is to be transferred to the statutory health insurance setting, the exercise bonus could easily be reactivated. Bonus systems are already in place that reward healthy behaviour and therefore could easily be used and adapted [80]. Due to the differences in social structure in the statutory system, the usage rate should also be higher. When investigating the effect of the exercise bonus, further research could split intervention groups in half or repeat the trial without an exercise bonus.

The chosen time horizon of this study is a strength as well as a limitation. Our study period of four years exceeds that of other published analyses, but it might still exclude savings that occur further down the line. Generali's business case before the implementation of the programme was calculated for five years, which suggests that more savings are expected later on. For a complex intervention like the one examined in the present study, the *Medical Research Council* recommends a lifetime horizon to demonstrate the sustainability of outcome changes [81]. Since no information was available on the self-assessed health status after the follow-up period, this could not be done in the present study. However, this evaluation is the first health cost-effectiveness study of its type in Germany: it was conducted using data obtained from a German private health insurance provider on a back pain-specific health intervention that was actually offered, implemented and followed up in the scope of an accompanying clinical trial. Therefore, this study was able to address the aforementioned uncertainty gap about the cost-effectiveness of long-term MBR.

### Policy implications

The present analysis can provide a blueprint for establishing disease management programmes for chronic low back pain in Germany. The German Federal Joint Committee (G-BA) published basic recommendations for the implementation of structured DMPs for CLBP in April 2019 [82]. Among other things, the G-BA recommended DMPs for participants with GCPS Grade II back pain and higher combined with at least 12 weeks of pain persistence. Moreover, participants should be motivated to be physically active. Interventions should be designed in such a way that patients are motivated to integrate the desired positive physical activity behaviour into their lifestyle in a self-responsible and sustainable way. In short, most of the G-BA recommendations are already part of the MBR investigated here.

The concrete implementation occurs regionally through the different sickness-funds of the statutory health insurance system [83]. The contents of back pain-specific DMPs in Germany are subject to negotiations between sickness funds and providers [26]. Each DMP must be approved by the Federal Office for Social Security [84]. The present analysis gives health decision-makers from sickness funds meaningful reference information regarding the expected budget impact of an exemplary DMP.

Based on the results of the present CEA, participants should be screened for their back pain severity at the beginning of an intervention and offered a DMP tailored to their needs. A positive budget benefit for the statutory health insurance system can be expected if the participants are treated according to their back pain severity. To guarantee cost savings in direct health costs, patients receiving the MBR programme investigated here should have severe chronic low back pain (Grade III onwards). Patients with minor back pain-related impairment should receive a different offering of services in order to achieve a dominant intervention.

The budget impact could be further improved by a high participation rate. On the one, hand the sickness fund would receive a financial incentive by the statutory risk structure compensation scheme for every enrolled participant (currently €145.44) [85]. On the other hand, large sickness funds should, in view of their size and expected number of participants, be able to negotiate more favourable terms with health care providers and thus reduce the costs of the individual interventions. A third option would be for insurance companies to establish their own training centres, as AOK Baden-Württemberg, a major German statutory health insurer, has done since 2005 (“AOK-RückenStudios”) [71]. Even though the initial implementation cost for this would be quite high, it could become a worthwhile investment if the sickness fund had a high market share of insured individuals near the training centre.

Proximity to the training location is important. A long distance from the place of residence to the nearest training centre was the most frequently cited reason for premature termination of the intervention [28]. This could be the main barrier to nationwide, large-scale implementation of the proposed MBR as well. Decision-makers should screen their customers for allocation and proximity to training centres by a provider very closely before deciding which one to choose.

A fully operating DMP for CLBP in Germany is long overdue. It has been proven that improved management of CLBP has the potential to achieve significant health improvements and direct and societal cost savings. We demonstrated that the MBR programme for chronic low back pain investigated here results in an excellent QALY gain, cost savings, and a substantial reduction in sick days. Hence, this cost-effective MBR can serve as a blueprint for establishing treatment programmes that comply with the clinical guidelines and recommendations of the G-BA and that are both medically and economically effective.

### Generalisability

The results of this study can be generalised to a limited extent. The data used was provided by a private health insurance company. Private health insurance members are generally healthier individuals than statutory health insurance members [86]. Thus, it can be assumed that the high improvement rate in back pain in the control group is also due to the participants’ good health insurance risk profiles. Good insurance risks are known to take care of their own health and believe in self-efficacy [87, 88]. The favourable effect of the investigated MBR might be higher in the general population, where a more passive control group can be expected.

With a gender ratio of 65 males to 35 females, this study had a surplus of males. This is not representative of the overall German population but can be considered representative of private health insurance populations [89]. To achieve better comparability with the statutory health insurance, costs for specific private insurance benefits (e.g., one or two-bed hospital rooms) were not considered. Additionally, the influence of the individual's tariff was kept as low as possible by using the billed amount for cost calculation and not the reimbursed amount.

Regarding the perspective of the analysis, this study was conducted from the payer's point of view. Indirect medical costs incurred by the individual, their relatives, society or the employer were not taken into account. Considering the apparent savings in sick days and the improvement in general health status, further savings beyond direct medical costs are conceivable.

The data used in this analysis was obtained from a single clinical trial, which potentially limits the generalisability of the findings for several reasons [90]. However, the fact that the study was multicentric and the participants and study centres were spread across Germany mitigated this factor.

The effects shown in this study using a relatively small sample size suggest that a participation in an outpatient MBR with behaviour-change coaching and device-supported exercise leads to cost savings, reduction of sick days as well as improvements in health status. The different effects were backed up with extensive sensitivity analyses, which increased the robustness of the findings. To erase the still prevailing doubt, an evaluation with a larger group is recommended.

## Conclusions

This is the first cost-effectiveness study to combine data from a private health insurance provider and a clinical controlled trial in Germany to demonstrate that long-term MBR is a cost-effective treatment for CLBP over a 24-month follow-up period. Patients with major impairment due to back pain, identified using Korff's Chronic Pain Grade Questionnaire, benefitted more from the intervention than those with minor impairment. Therefore, we conclude that MBR should be recommended for patients with major impairment due to back pain. Another solution needs to be developed for those with minor impairment. Health insurance providers should use their administrative data to identify individuals with back pain-related impairment so that they can offer the appropriate services in a targeted manner. It should be stressed that MBR significantly reduced sick leave in both groups of patients with minor or major impairment over the long term. Hence, MBR can be characterised as a profitable intervention from societal point of view. However, it remains to be seen how the cost-effectiveness curves for MBR will develop over the lifetime horizon.

## Data Availability

The data supporting the findings of this study are available from Generali Deutschland Krankenversicherung AG and the University of Lübeck on request. However, some restrictions apply to the availability of these data, which were used under license for the current study and therefore are not publicly available. The data is however available from the authors upon reasonable request and with permission, including a signed data access agreement from Generali Deutschland Krankenversicherung AG and the University of Lübeck. Data management and statistical analyses were carried out using the software R with the application of the packages listed in the bibliography. The code that supports the findings of this study is available from Generali Deutschland Krankenversicherung AG, but restrictions apply. It is available from the authors upon reasonable request and with the necessary permissions, including a signed access agreement from Generali Deutschland Krankenversicherung AG.

## Abbreviations

ATT: Average effect of treatment on the treated
CCI: Charlson’s Comorbidity Index Score
CEA: Cost-effectiveness analysis
CHEERS: Consolidated Health Economic Evaluation Reporting Standards
CI: Confidence Interval
CLBP: Chronic lower back pain
DiD: Difference-in-difference
DMP: Disease management programmes
FPZ: Cologne Research and Prevention Centre
G-BA: German Federal Joint Committee
GCPS: Graded Chronic Pain Scale
HRQOL: Health-related quality of life
ICD-10: 10th revision of the International Statistical Classification of Diseases and Related Health Problems
ICER: Incremental cost-effectiveness ratio
IQWIG: German *Institute for Quality and Efficiency in Health Care*
ITT: Intention-to-treat
MBR: Multidisciplinary biopsychosocial rehabilitation
NICE: National Institute for Health and Care Excellence
PHQ-4: Patient Health Questionnaire 4
PSM-DiD: Propensity score matching difference-in-difference
QALY: Quality-adjusted life year
SE: Standard error
SF-12: Short Form 12 health survey instrument
SHI: Statutory Health Insurance
WTP: Willingness to pay
YLD: Years lived with disability

## Footnotes

1 Invitations were not controlled for the distance between the FPZ training centre and the patient’s home. Therefore, some participants only realised after enrolling that the distance to reach the training centre was not manageable on a regular basis. These participants did not drop out because of their pain levels but because of access problems. The percentages of dropouts were comparable (4.7 and 6.6%) in the respective pain groups.
